# Profiling of N6-methyladenosine methylation in porcine *longissimus dorsi* muscle and unravelling the hub gene *ADIPOQ* promotes adipogenesis in an m^6^A-YTHDF1–dependent manner

**DOI:** 10.1186/s40104-023-00833-4

**Published:** 2023-04-06

**Authors:** Huanfa Gong, Tao Gong, Youhua Liu, Yizhen Wang, Xinxia Wang

**Affiliations:** 1 Key Laboratory of Molecular Animal Nutrition, Ministry of Education, College of Animal Sciences, Zhejiang University, Hangzhou, 310058 People’s Republic of China; 2 Key Laboratory of Animal Nutrition and Feed Science in Eastern China, Ministry of Agriculture, College of Animal Sciences, Zhejiang University, Hangzhou, 310058 People’s Republic of China

**Keywords:** ADIPOQ, Intramuscular fat, N6-methyladenosine, Pig, YTHDF1

## Abstract

**Background:**

Intramuscular fat (IMF) content is a critical indicator of pork quality, and abnormal IMF is also relevant to human disease as well as aging. Although N6-methyladenosine (m^6^A) RNA modification was recently found to regulate adipogenesis in porcine intramuscular fat, however, the underlying molecular mechanisms was still unclear.

**Results:**

In this work, we collected 20 *longissimus dorsi* muscle samples with high (average 3.95%) or low IMF content (average 1.22%) from a unique heterogenous swine population for m^6^A sequencing (m^6^A-seq). We discovered 70 genes show both differential RNA expression and m^6^A modification from high and low IMF group, including *ADIPOQ* and *SFRP1*, two hub genes inferred through gene co-expression analysis. Particularly, we observed *ADIPOQ*, which contains three m^6^A modification sites within 3′ untranslated and protein coding region, could promote porcine intramuscular preadipocyte differentiation in an m^6^A-dependent manner. Furthermore, we found the YT521‑B homology domain family protein 1 (YTHDF1) could target and promote *ADIPOQ* mRNA translation.

**Conclusions:**

Our study provided a comprehensive profiling of m^6^A methylation in porcine *longissimus dorsi* muscle and characterized the involvement of m^6^A epigenetic modification in the regulation of *ADIPOQ* mRNA on IMF deposition through an m^6^A-YTHDF1-dependent manner.

**Supplementary Information:**

The online version contains supplementary material available at 10.1186/s40104-023-00833-4.

## Background

Intramuscular fat (IMF) content is a critical indicator in pork consume, and also linked to insulin resistance [1], aging [2] and obesity [3] in human. Pig works as an ideal human biomedical model with advantage over primates and other livestock due to its high similarities with human being from the anatomy and physiology [4]. Therefore, illustrating the molecular mechanism underlying the IMF deposition is vital for pork consumption and human health.

N6-Methyladenosine (m^6^A) is the most prevalent post-transcriptionally modification in eukaryotic cells, emerging as an important epigenetic regulator in various physiological processes [5, 6]. Dynamic mRNA m^6^A modification is regulated by dedicated methyltransferases (“writers”) and demethylases (“erasers”) [7]. RNA-binding proteins (“readers”) could recognize m^6^A-containing transcripts to drive RNA processes [8, 9], such as mRNA stability [9], splicing [10] or translation [11]. For instance, YT521‑B homology domain family protein 1 (YTHDF1) promotes breast cancer metastasis via enhancing FOXM1 translation in an m^6^A-dependent manner [12]. Fat mass and obesity-associated (FTO) protein regulates the splicing of adipogenic regulatory factor RUNX1T1 through affecting m^6^A level around splice site [13]. It has been reported that m^6^A is highly enriched around the stop codons and 3’UTRs [5]. Recent progress also indicated that m^6^A methylation of the 3’UTR of FLC causing depletion of its mRNA, controlling flowering in *Arabidopsis* [14].

Accumulating evidences suggested that m^6^A modification played important roles in regulating various aspects of mRNA metabolism during adipose tissue expansion [15–18]. For instance, NADP modulates m^6^A methylation and adipogenesis by enhancing FTO activity in 3 T3-L1 preadipocytes [19]. Consistently, Zfp217 mediates mRNA m^6^A methylation through FTO and YTHDF2 to regulate adipogenesis [20]. Furthermore, m^6^A modification of two adipogenesis-related genes, *UCP2* and *PNPLA2*, would both regulate adipogenesis between Chinese indigenous breed Jinhua (fatty) and Western commercial breed Landrace (lean) in backfat, whereas in an opposite way [21]. Although it has been reported that YTHDF1 directly targets MTCH2 to promote adipogenesis in porcine intramuscular preadipocytes, our understanding about the function of m^6^A modification in IMF deposition was still limited.

Here we aimed to provide a valuable resource to determine the effects of m^6^A modified genes potentially involving in adipogenesis of IMF, permitting us to better understanding how to improve pork quality and providing potential target for therapy of obesity.

## Materials and methods

### Animal, phenotype and sample collection

This study utilized a mosaic swine population to uncover the relationship of m^6^A regulation mechanism and IMF deposition. The heterogeneous pig stock was derived from eight founder breeds (F0) consisting of the four Western commercial breeds (Duroc, Large White, Landrace and Pietrain pigs) and the four Chinese indigenous breeds (Erhualian, Laiwu, Bamaxiang and Tibetan pigs). All the pigs were raised under the same condition and purposeful mating, crossbreed strategy in detail was described previously [22, 23]. Animals were slaughtered in commer abattoir at 240 ± 10 d. We selected the *longissimus dorsi* muscle (LDM) from the 6^th^ generation (F6; average IMF: 2.28%, range 0.92%–7.45%) [23]. LDM was obtained between the 3^rd^ and 4^th^ lumbar vertebrae, and flash frozen in liquid nitrogen and stored at −80 °C before use. The intramuscular fat content was measured using the routine Soxhlet extraction method [24].

Intramuscular preadipocytes cells were isolated from the LDM of 3-day-old Duroc-Landrace-Yorkshire piglets under sterile conditions [15]. The experimental procedures were in compliance with guidelines of the Committee on Animal Care and Use and Committee on the Ethic of Animal Experiments of Zhejiang University (Hangzhou, China).

### RNA extraction and m^6^A RNA immunoprecipitation sequencing

Total RNA was isolated and purified using Trizol reagent (Invitrogen, Carlsbad, CA, USA) refer to the instruction, criteria with RIN > 7.0, total RNA > 50 μg, concentration > 50 ng/μL and OD_260__/__280_ > 1.8 were left for subsequent use. Poly (A) RNA is purified from 50 μg total RNA using Dynabeads^TM^ Oligo (dT)_25_–61005 (Thermo Fisher Scientific Baltics UAB; Vilnius, Lithuania) using two rounds of purification. Then the poly(A) RNA was fragmented into small pieces using Magnesium RNA Fragmentation Module (NEB, cat.e6150, USA) under 86 °C for 7 min.

Approximately 50 ng of fragmented mRNA was saved as input sample, which was used to eliminate the background. m^6^A-sepecific methylated RNA sequencing was performed according to the previous report [25]. In brief, the other fragmented mRNA was incubated with 3 μg methylated RNA-specific antibodies (No. 202003, Synaptic Systems, Göttingen, Germany) in RIP buffer (150 mmol/L NaCl, 10 mmol/L Tris and 0.1% NP-40) at 4 °C. After 2 h, adding the washed protein A/G magnetic beads (Millipore, Billerica, MA, USA) and incubating at 4 °C for further 2 h. Beads, washed 6 times in RIP buffer, incubated with immunoprecipitation buffer (Sigma-Aldrich, St Louis, MO, USA) to elute RNA. Immunoprecipitated RNA was extracted with phenol/chloroform, and RNA samples were sent for next-generation sequencing. All libraries were sequenced for 150 bp paired-end sequencing under an Illumina Novaseq™ 6000 (LC-Bio Technology CO., Ltd., Hangzhou, China) following the vendor’s recommended protocol.

### Quantitative of m^6^A level by liquid chromatography-tandem mass spectrometry (LC-MS/MS)

Quantification of m^6^A in mRNA was conducted based on the previous study [26]. In brief, 300 ng of mRNA was digested by nuclease P1 (2 U) at 42 °C for 2 h, followed by the addition of alkaline phosphatase (0.5 U) with incubation at 37 °C for 2 h. The total amount of m^6^A in RNA was measured using Waters Acquity UPLC coupled to a Waters Xevo TQ mass spectrometer (Waters, Milford, USA). Quantification was achieved by comparing with the standard curve obtained from pure nucleoside standards. The ratio of m^6^A to A was calculated based on the determined concentration.

### RNA mapping and quality control

Raw data were evaluated with FastQC v0.11.9 [27], the heading 10 bp were removed using trimmomatic v0.39 on account of GC bias [28]. Clean data were mapped to *Sus scrofa* 11.1 using STAR v2.7.8a, SAMtools v1.11 was used for sorting and marking duplicated reads [29, 30]. IP data were performed the same mapping procedure as input data.

### MeRIP-seq data analysis

For IP data, m^6^A peak calling was conducted by MACS2 with “--nomodel -g 2.5e9 --broad --keep-dup all” on whole transcript level. Differentially peaks were identified with in-house R script according to previous study [31, 32]. Briefly, bedtools was used to combine all peaks from High and Low group into a reference peak. Normalized depth of each peak was inferred by following method: Normarlized depth = ((IP reads of Peak Region/Total reads of IP sample) − (Input reads of Peak Region/Total reads of input sample))/Length of peak. Total number of each sample’ read was calculated by SAMtools v1.11 flagstats based on BAM file. Coverage of peaks were inferred using SAMtools v1.11 bedcov.

Differentially methylated peaks (*P* < 0.05 and abs (log_2_foldchange ) > log_2_1.5) were identified by comparing average normalized depth of each peak between High and Low group using *t*-test in R program. VEP software was using for annotating the differential peaks. HOMER software was applying for uncovering the motif in conserved peak regions.

### Input data analysis

Input data were used for annotating, merging and quantifying with StringTie v2.1.7. raw counts of transcripts then were normalized (described in the legend of Fig. 3f) and low expression genes (gene counts > 9 in less than 4 samples) were filtered. Differential expression transcripts/genes were uncovered by the DESeq2 software [33, 34]. (abs (log_2_foldchange )) > log_2_1.5 and *P* < 0.05 were identified as differentially expressing transcripts/genes.

Principal component analysis was conducted with DESeq2 [33]. Briefly, high expression gene counts were used for constructing DESeq data with the function *DESeqDataSetFromMatrix*(). And then data was normalized by the function *rlogTransformation*(). PCA was inferred with the function *plotPCA*() and visualized in R program. Pheatmap package was performed for visualing heatmap.

### Weighted gene co-expression network analysis

Co-expression network analysis was performed with WGCNA (Wegithed Gene Co-expression Network Analysis) R package [35]. Briefly, raw count of genes were infered from the input data, and then low expression genes (gene counts > 9 in less than 4 samples) were filtered. Reserved gene counts were normalizd with transcript per millon (TPM) method. The soft threshold power β is determined based on the standard scale-free network, inferred from the function *pickSoftThreshold()*.The adjacency matrix was calculated using topological overlap measure (TOM) [36], hierarchically clustering coexpressed genes into modules. Module-trait associations were calculated as the Pearson’s correlation between the module eigengene and trait of interest [37]. The most relevant traits of module was selected for analyzing their biological function and uncovering hub genes. Hub genes are a group of genes with the highest connectivity, and determine the characteristics of the gene module. We defined hub genes which are the significant correlation with clinical characteristics (Gene Significance, GS > 0.2) and high module characterization (Module Membership, MM > 0.8) in the module.

### Functional enrichment analysis

Gene Ontology (GO) and Kyoto Encyclopedia of Genes and Genomes (KEGG) pathway enrichment analyses were conducted by ClueGO in Cytoscape v3.9.0. Pathways with *P* ≤ 0.05 were selected, *P*-value was chosen from the term *P*-value corrected with Bonferroni step down. GO ontologies involve biological process, cellular component and molecular function.

### Western blot analysis

Cells were lysed with the mixture containing cell lysis buffer for Western and IP and 1% phenylmethanesulfonyl fluoride (PMSF) (Biosharp, Beijing, China) on ice to extract protein. Protein samples were separated by SDS-PAGE and then transferred to polyvinylidene difluoride membranes. And the membranes were blocked with 5% non-fat milk at room temperature for 1 h, then incubated with the primary antibody overnight at 4 °C and next with the secondary antibody for 1 h at room temperature. The protein bands were visualized using ECL Protect from Light (Biosharp) and quantified using Image J software. The primary antibodies used in this study were as follows: ADIPOQ (sc-136131, Santa Cruz, Watsonville, CA, USA, diluted 1:200), FLAG (20543–1-AP, Proteintech, Rosemont, IL, USA, diluted 1:1,000), YTHDF1 (17479–1-AP, Proteintech, diluted 1:1,000), GFP (ET160–25, Huabio, Hangzhou, China, diluted 1:5,000), β-actin (M1210–2, Huabio, diluted 1:5,000). The secondary antibodies were as follows: goat anti-mouse IgG-HRP (HA1006, Huabio, diluted 1:2,000), goat anti-rabbit IgG-HRP (HA1001, Huabio, diluted 1:2,000).

### Real-time quantitative PCR (qPCR) analysis

Total RNA was extracted using TRIzol (Biosharp) according to the product protocol. After examination of RNA purity and concentration, 2 μg RNA was used as a template to reverse transcribe to cDNA by using M-MLV Reverse Transcriptase Kit (Invitrogen). Reverse transcription conditions were under 5 min at 25 °C, 45 min at 50 °C, 5 min at 85 °C. qPCR analysis was performed using the SYBR Green PCR Master Mix (Roche, Basel, Switzerland) with the ABI Step-One PlusTM Real-Time PCR System (Applied Biosystems, Waltham, MA, USA). Relative level of RNA expression was determined with 2^−ΔΔCt^ method after normalization to *GAPDH*. Reaction conditions were 95 °C for 1 min, 40 cycles of 95 °C for 15 s and 60 °C for 30 s. Primers used in this study were listed in Table 1.

**Table 1:** Primer sequences used in this work

Name	Forward primer (5′→3′)	Reverse primer (5′→3′)
*ADIPOQ*	TATGATGTCACCACTGGCAAA	TAGAGGAGCACAGAGCCAGAG
*PPARγ*	AGGACTACCAAAGTGCCATCAAA	GAGGCTTTATCCCCACAGACAC
*CEBPβ*	GCACAGCGACGAGTACAAGA	TATGCTGCGTCTCCAGGTTG
*aP2*	CAGGAAAGTCAAGAGCACC	ATGATACATTCCACCACCAA
*GAPDH*	ACACTCACTCTTCCACTTTTG	CAAATTCATTGTCGTACCAG

### Oil Red O staining

Oil Red O staining was performed as following procedures: cells were washed and fixed with 10% formalin for 1 h, and then washed 3 times with 60% isopropanol. Cells were stained with Oil Red O working solution (0.35% Oil Red O dye in 60% isopropanol) for 10 min, and further washed 4 times with distilled water. Cells were eluted the stained lipid droplets using 100% isopropanol for 10 min, and then measuring optical density (OD) at 500 nm to conduct the quantitative of lipid content.

### Intramuscular preadipocytes (IMF cells) isolation

IMF cells were isolated based on the previous study [15]. Briefly, the LDM of 3-day-old Duroc-Landrace-Yorkshire piglets were separated under sterile conditions. Visible connective tissue was removed and finely minced. Muscle tissues were then digested in a digestion buffer consisting of 1 mg/mL collagenase type I (Gibco, Carlsbad, CA, USA) in a shaking water bath for 1.5 h at 37 °C. The digested sample was filtered aseptically through 80 and 200 μm nylon mesh filters to isolate cells. Filtered cells were then washed 3 times with Dulbecco’s Modified Eagle Medium (DMEM) via centrifugation at 1,500 r/min for 5 min. Cells were seeded in growth medium that consisted of DMEM medium containing 10% fetal bovine serum (Gibco) and 1% penicillin-streptomycin (Gibco). After 1 h, cells were rinsed with DMEM medium to remove unadhered cells, and the adhered cells consisted of pure IMF cells.

### Cell culture and adipocyte differentiation

Cells were cultured in DMEM containing 10% fetal bovine serum (Gibco) and 1% penicillin-streptomycin (Gibco). At 2 d after confluence, defined as d 0, cells were induced to differentiation medium containing 0.5 mmol/L 3-Isobutyl-1-methylxanthine (IBMX), 1 μmol/L dexamethasone and 5 μg/mL insulin (Sigma, St. Louis, MO, USA). On d 2, the medium was replaced with maintenance medium containing 5 μg/mL insulin (Sigma) every 2 d until d 8. Two hundred and ninety-three T cells were cultured in DMEM/F12 medium containing 10% fetal bovine serum and 1% penicillin-streptomycin (Gibco). Cells were uniformly cultured in a 5% CO_2_ incubator with 37 °C.

### Cell transfection, plasmids and RNA knockdown

The plasmids and siRNA transfections were performed using Hieff Trans™ Liposomal Transfection Reagent and Hieff Trans™ in vitro siRNA/miRNA Transfection Reagent (Yeasen, Shanghai, China), according to the product protocol. The adenoviruses ADV4-ADIPOQ-CDS wild-type (ADV4-ADIPOQ-CDS-WT), ADV4-ADIPOQ-CDS mutant (m^6^A C_534_ and C_570_ were replaced by T, ADV4-ADIPOQ-CDS-MUT) and ADV4-ADIPOQ-CDS negative control (ADV4-ADIPOQ-CDS-NC) were generated by GenePharma (Shanghai, China). IMF cells were infected with the multiplicity of infection (MOI) of 25:1 by ADV4-ADIPOQ-CDS-WT, ADV4-ADIPOQ-CDS-MUT and ADV4-ADIPOQ-CDS-NC, respectively, and added 1 μg/mL polybrene to improve the infection efficiency, according to GenePharma’s protocol. Porcine YTHDF1 cDNA was generated via PCR and cloned into the pFLAG-CMV2 expression plasmid. Sequences of siRNA, synthesizd by GenePharma (Shanghai, China), were as follows: siADIPOQ-F, 5′- AGAAAGCGCCUAUGUCUACTT-3′ and siADIPOQ-R, 5′-GUAGACAUAGGCGCUUUCUCC-3′; siYTHDF1-F, 5′-UUAGUAUCCUGUCCUUUUGUU-3′ and siYTHDF1-R, 5′-CAAAAGGACAGGAUACUAAAG-3′.

### m^6^A-specific methylated RNA immunoprecipitation real-time PCR

m^6^A-qPCR analysis was conducted according to previously report [38]. Briefly, mRNAs fragmented by RNA fragmentation reagent (Invitrogen) at 70 °C for 15 min. 10% of fragmented RNAs was used as input control mRNAs. The remaining 90% was immunoprecipitated with anti-m^6^A antibody coupled to Dynabeads (Invitrogen) in immunoprecipitation buffer (RNase inhibitor, 10 mmol/L Tris-HCl, 150 mmol/L NaCl, 0.1% Igepal CA-630 [Sigma]) at 4 °C for 2 h. mRNAs containing m^6^A were eluted twice with m^6^A 5′-monophosphate sodium salt (Sigma) at 4 °C for 1 h. After ethanol precipitation, all mRNAs were reversely transcribed into cDNA by M-MLV reverse transcriptase (Invitrogen). And then m^6^A enrichment was determined by qPCR. Data were analyzed with the 2^−ΔΔCt^ method, and the relative enrichment of m^6^A in each sample was calculated by normalizing to input. The primers were as follows: ADIPOQ-CDS-F, 5′- TCCTTCCACATCACGGTCTACT-3′ and ADIPOQ-CDS-R, 5′- CTCCAGATAGAGGAGCACAGAG-3′; ADIPOQ-3’UTR-F, 5′-CCACTGTGTTTCCTCAGGTTC-3′ and ADIPOQ-3’UTR-R, 5′- CCACAGCCCTGTGTTTGACTT-3′.

### RNA immunoprecipitation assay

The experiment pipeline was performed according to the previous research [39]. Briefly, FLAG-YTHDF1 overexpressed IMF cells were lysed in lysis buffer for 30 min at 4 °C and the supernatant was collected for further use. We saved 50-μL aliquot of cell lysate as input, and the remaining was incubated with anti-FLAG or immunoglobulin G (IgG) antibody-conjugated magnetic beads (Sigma) for 4 h at 4 °C. The beads were washed with buffer containing 0.1% SDS and proteinase K (Invitrogen), detecting fold enrichment with qPCR.

### Dual-luciferase reporter and mutagenesis assays

To evaluate the effect of 3’UTR m^6^A site on ADIPOQ expression, wild type or mutant (m^6^A A_650_ was replaced by T) of ADIPOQ-3’UTR was inserted into downstream of pmirGLO Dual-Luciferase vector (Promega, Madison, WI, USA). After 48 h post transfection, the activities of firefly luciferase and Renilla luciferase in each 24-well plates’ well were determined by a Dual-Luciferase Reporter Gene Assay Kit (Yeasen) according to the product protocol.

### Statistical analysis

All data were presented as mean ± SEM. Statistical differences in the dual luciferase reporter assay were determined by Mann-Whitney test, and other statistical significance between multiple groups were determined by Student’s *t*-test with GraphPad Prism 9. *P* < 0.05 was considered exceeding the significant level.

## Results

### Description of m^6^A modification between high and low IMF content groups

**Fig. 1:**
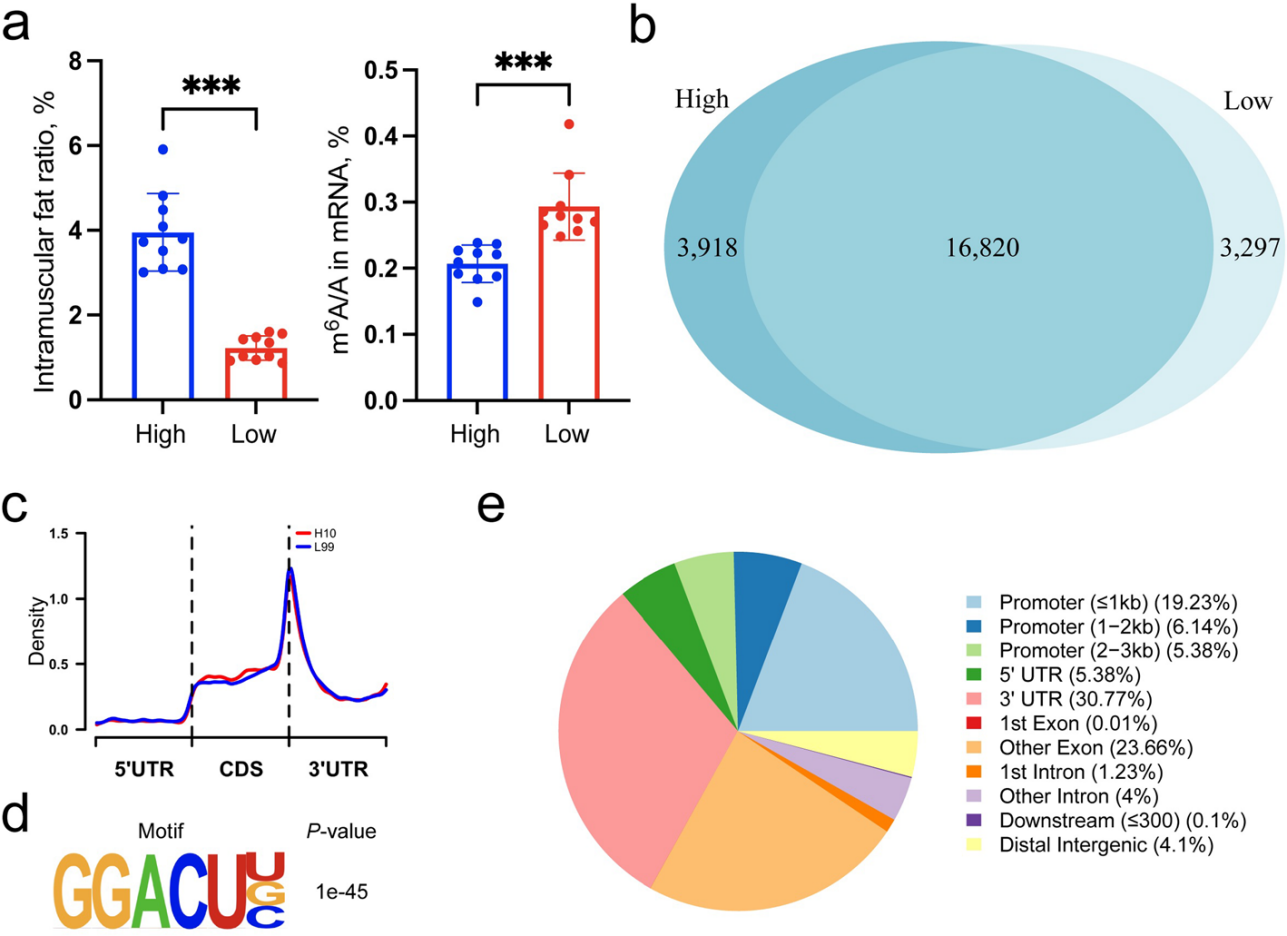
Overview of m^6^A modification in High and Low IMF content groups. **a** Intramuscular fat ratio (right) and m^6^A/A content (left) among High and Low group, *n* = 10. **b** Venn diagram of peaks among two groups. **c** Density of m^6^A modification across mRNA region. **d** Conserved motif in m^6^A peaks using HOMER software. **e** Annotation of location of m^6^A peak at whole-transcript level. ^***^P < 0.001

To investigate the role of m^6^A modification on adipogenesis in LDM, we collected 20 extreme phenotypic samples of IMF content from the 6^th^ generation individuals in a unique heterogeneous swine population, which exhibits a large variation of IMF content [22, 23]. The samples were divided into high and low group according to IMF content (High and Low), and LC-MS/MS was performed to evaluate the m^6^A modifications levels across the samples. We found that IMF content (left in Fig. 1a) and level of m^6^A modifications (right in Fig. 1a) displayed opposite trend across the group, while both of those were significantly divergent among High and Low (*P* < 0.01), in agreement with previous study [15]. We uncovered 20,738 and 20,117 peaks among High and Low (Fig. 1b), respectively. A total of 23,250 peaks as a m^6^A modification panel within this population were obtained by “bedtools merge -d 0”. Conserved m^6^A modification motif among the panel was concordance with previous study (RRACH (R = G or A and H = A, C or U)) using HOMER (Fig. 1d). m^6^A modification sites were accumulating at the stop codon site (Fig. 1c) [15]. Peaks, annotated with ChIPseeker, were mainly enriched in the 3’UTR (Fig. 1e). These results together suggested our data was credible to further investigate the effect of m^6^A modification on lipid deposition in LDM.

### Identifying co-expression gene module of LDM

**Fig. 2:**
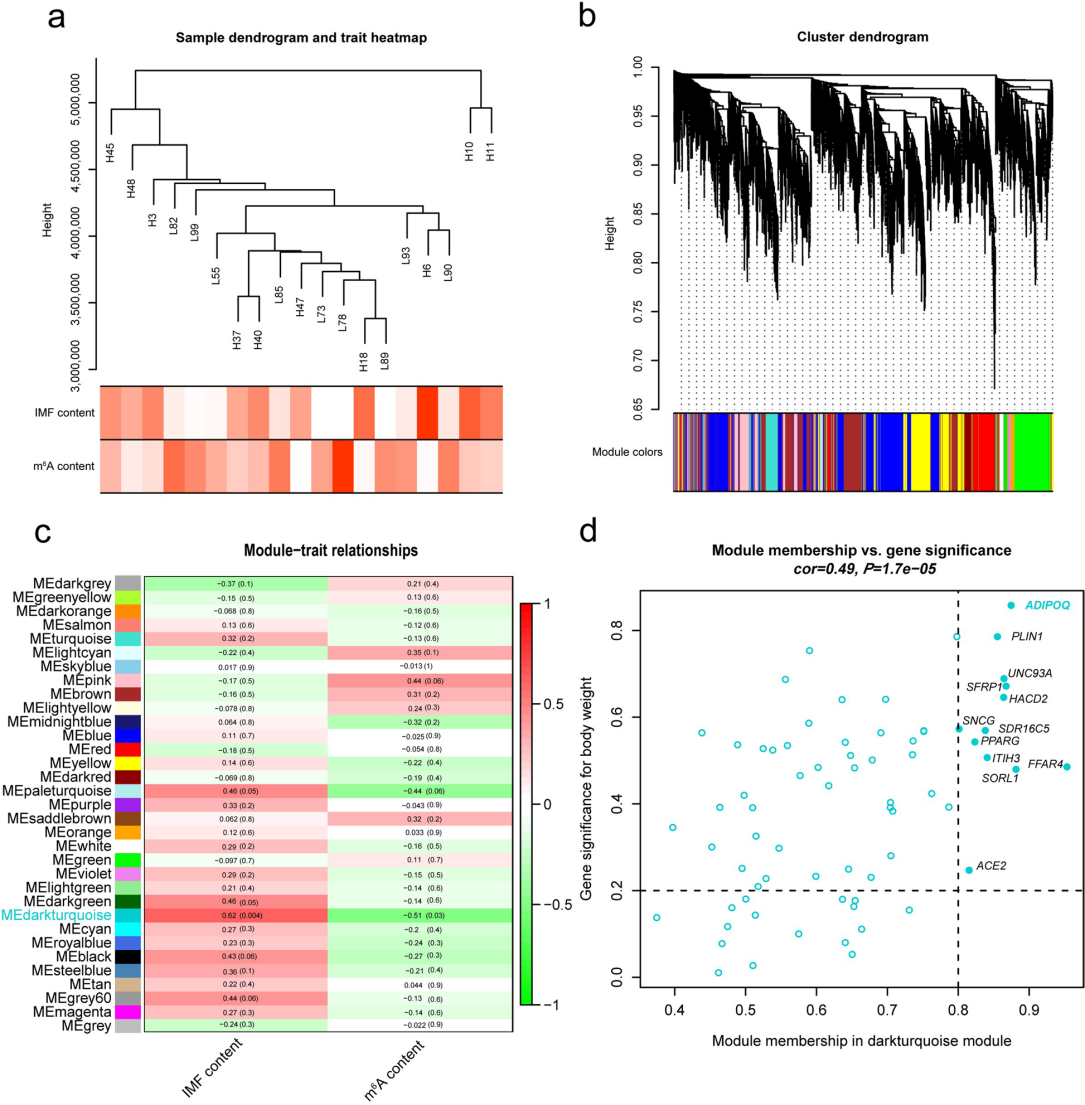
Network analysis of MeRIP-seq input data of LDM samples. **a** Sample dendrogram from 19 LDM samples and trait heatmap including content of IMF and m^6^A modifications. Color intensity is directly proportional to the value of corresponding trait. **b** Cluster dendrogram of 13,245 highly expressing genes. Thirty-three co-expression modules were identified, each color represents a module. **c** Heatmap of the correlation between module eigengenes (MEs) and traits. Left value is correlation, and right enclosed in bracket is *P*-value. **d** Scatter plot for 70 genes in dark turquoise module, gene significance (GS) > 0.2 and module membership (MM) > 0.8 were selected as hub gene

Co-expression network analysis enable us to identify genes which have a tendency to show a coordinated expression pattern among samples, uncovering the complexity of a cellular transcription network [37, 40]. Thus, we conducted the WGCNA software [35] to construct a co-expression network with 13,245 highly expressing genes (≥ 10 reads in at least 16 individuals) among 19 samples from the input RNA sequencing (RNA-seq) data of m^6^A-seq. L96 was excluded for outlier clustering according to the PCA and heatmap (Fig. S1a–d). We then chosen the optimal weighting coefficient β = 7 to construct the network based on pickSoftThreshold parameter in WGCNA. Figure 2a shows the cluster tree of the 19 samples and the corresponding traits information. Of 33 identified gene modules (Fig. 2b), MEdarkturquoise (module eigengene in dark turquoise) with 70 genes (Fig. 2c; Table S1) was detected significantly positively related to IMF content (*r* = 0.62; *P* = 0.004) and highly negatively associated with m^6^A content (*r* = − 0.51; *P* = 0.03) respectively, suggesting these genes within the module potentially participant in fat deposition. To investigate the underlying role of these co-expression genes, we performed the KEGG and GO pathway enrichment analysis by ClueGO in Cytoscape v3.9.0 [41]. The significant biological processes were involved in several adipogenesis related pathways, such as regulation of fat cell differentiation (*ADIPOQ*, *BMP2*, *CEBPα*, *PPARγ*, *SFRP1*), positive regulation of fat cell differentiation (*BMP2*, *CEBPα*, *PPARγ*, *SFRP1*) and PPAR signaling pathway (*ADIPOQ*, *PLIN1*, *PPARγ*) (Table S5). In addition, we identified 12 hub genes (*ADIPOQ*, *PLIN1*, *UNC93A*, *SFRP1*, *HACD2*, *SNCG*, *SDR16C5*, *PPARγ*, *ITIH3*, *FFAR4*, *SORL1* and *ACE2*) from the dark turquoise module based on |geneModuleMembership| > 0.8 and |geneTraitSignificance| > 0.2 (Table 2). Of these, *ADIPOQ*, *PLIN1*, *SFRP1*, *PPARγ* and *FFAR4* have been reported to participate in adipogenesis related function [42–45]. Remarkably, *ADIPOQ*, *PLIN1* and *FFAR4* were identified higher expression in subcutaneous fat and intramuscular fat compared with LDM among the same heterogeneous swine population [23], hinting these hub genes may play critical roles in adipogenesis.

**Table 2:** Hub genes screened with WGCNA in porcine LDM

Gene stable ID	Gene name	MMvalue	GSvalue	Gene description
ENSSSCG00000039103	*ADIPOQ*	0.874557699	0.858025625	Adiponectin, C1Q and collagen domain containing [Source:VGNC Symbol; Acc:VGNC:85140]
ENSSSCG00000001844	*PLIN1*	0.855238258	0.785834744	Perilipin 1 [Source:VGNC Symbol; Acc:VGNC:91557]
ENSSSCG00000027404	*UNC93A*	0.864469943	0.688918599	unc-93 homolog A [Source:VGNC Symbol; Acc:VGNC:94711]
ENSSSCG00000025822	*SFRP1*	0.867467057	0.671675851	Secreted frizzled related protein 1 [Source:VGNC Symbol; Acc:VGNC:95493]
ENSSSCG00000034786	*HACD2*	0.864057374	0.645773129	3-hydroxyacyl-CoA dehydratase 2 [Source:VGNC Symbol; Acc:VGNC:88766]
ENSSSCG00000026850	*SNCG*	0.801110493	0.572908619	Synuclein gamma [Source:NCBI gene (formerly Entrezgene); Acc:100125343]
ENSSSCG00000006245	*SDR16C5*	0.838140788	0.569356922	Short chain dehydrogenase/reductase family 16C member 5 [Source:VGNC Symbol; Acc:VGNC:98853]
ENSSSCG00000011579	*PPARγ*	0.823399895	0.542931625	Peroxisome proliferator activated receptor gamma [Source:VGNC Symbol; Acc:VGNC:91684]
ENSSSCG00000011451	*ITIH3*	0.840818259	0.506913162	Inter-alpha-trypsin inhibitor heavy chain 3 [Source:VGNC Symbol; Acc:VGNC:89248]
ENSSSCG00000010478	*FFAR4*	0.953347586	0.485423388	Free fatty acid receptor 4 [Source:VGNC Symbol; Acc:VGNC:107392]
ENSSSCG00000015135	*SORL1*	0.88145227	0.479634802	Sortilin related receptor 1 [Source:HGNC Symbol; Acc:HGNC:11185]
ENSSSCG00000012138	*ACE2*	0.815256029	0.246877132	Angiotensin converting enzyme 2 [Source:VGNC Symbol; Acc:VGNC:85008]

*MMvalue* Value of Module Membership, the correlation of the module eigengene and the gene expression profile; *GSvalue* Value of Gene Significance, the absolute value of the correlation between the gene and the trait

### *ADIPOQ* gene display significantly difference in both m^6^A modification and RNA expression between high and low group

**Fig. 3:**
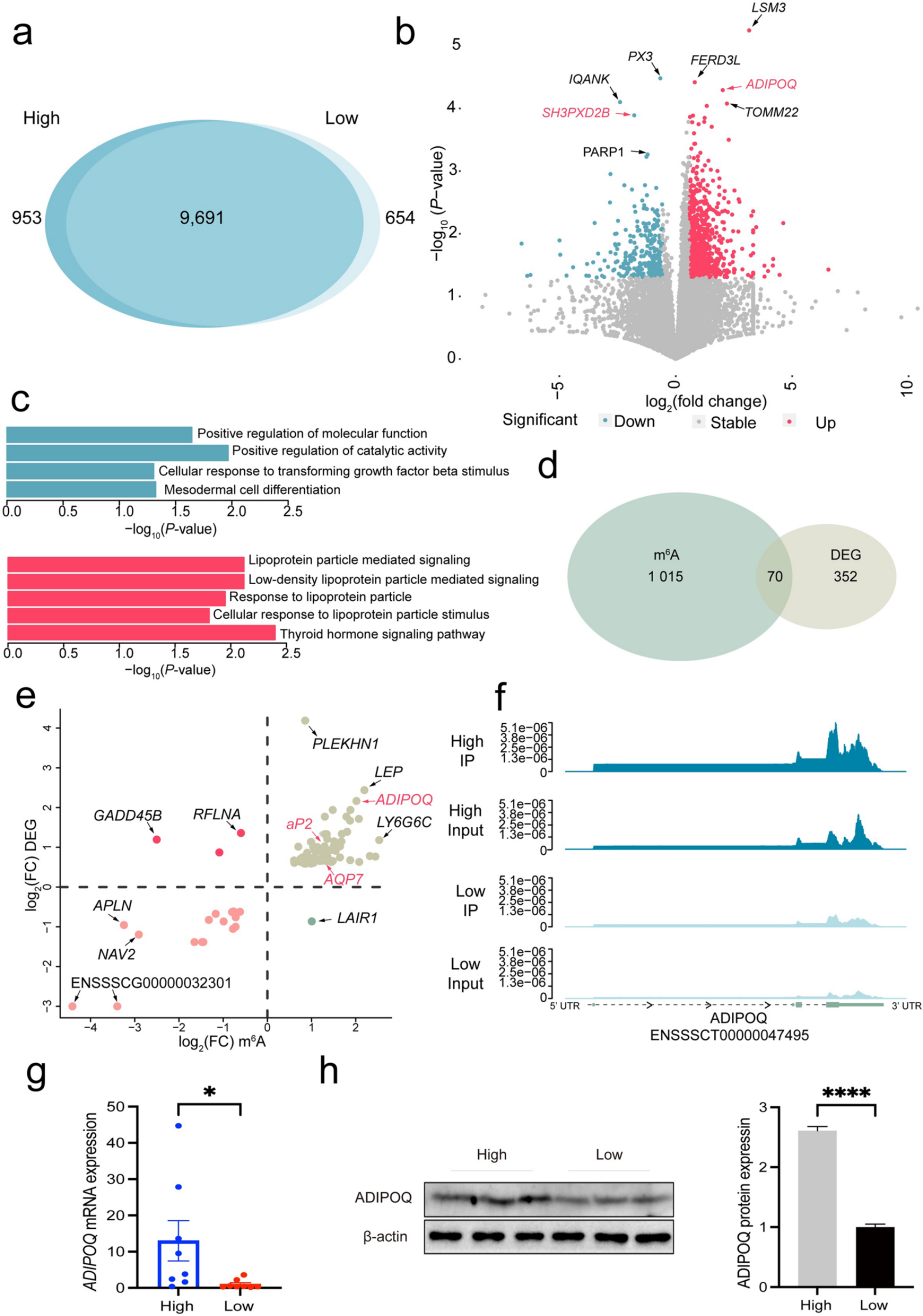
RNA expression differentially and m^6^A modification differentially genes between High and Low. **a** Venn diagram of m^6^A modified genes across High and Low groups. **b** Volcano plot of m^6^A modified differential gene, *P* < 0.05 and fold change > 1.5 were marked as differentially methylated genes (blue and red), fold change value is calculated by High/Low. **c** GO and KEGG pathways of down (blue) and up (red) regulated m^6^A modification genes. **d** Venn diagram and (**e**) four quadrant diagram of methylated and RNA expression differential genes (*P* < 0.05 and fold change > 1.5) between High and Low group, 70 genes were observed significantly co-differential in **e**. **f** and** g** m^6^A methylation and mRNA expression of *ADIPOQ* gene between High and Low group, *n* = 8. Normalized read count was employed for comparing the level of m^6^A methylation between High and Low. Normalized read Count = SRN/ITR, SRN is site of reads number, while ITR is individual of total reads. SRN was counted using SAMtools v1.11 bedcov, ITR was inferred using SAMtools v1.11 flagstats based on BAM file. Input and IP data were both under the same pipeline of normalization. **h** Protein level of ADIPOQ between High and Low, *n* = 3

To determine the role of m^6^A modification in intramuscular fat, we annotated the differential peak regions: 953 and 654 genes (Fig. 3a) were uniquely modified with m^6^A across the High and Low, respectively. One thousand and eighty-five genes (Fig. 3b; Table S2) were identified for significantly differential modified (abs (log_2_foldchange) > log_2_1.5; *P* < 0.05). Gene ontology analysis of these m^6^A modified regions were significantly enriched in lipoprotein related functions (Fig. 3c), suggesting mRNA m^6^A in *longissimus* muscle play a potential role in regulating fat deposition. Among the 8 top significant differentially modified genes (according to *P*-value), we observed *ADIOPQ* (*P* = 5.09E−05) and *SH3PXD2B* (*P* = 1.28E−04) were reported to regulate the fat cell differentiation (Fig. 3b) [46, 47]. Similarly, we discovered 422 differential expression genes (abs (log_2_foldchange) > log_2_1.5; *P* < 0.05) among the High and Low based on input RNA-seq data from m^6^A-seq (Fig. S1e; Table S3). Gene enrichment analysis revealed lipid droplet (*CIDEC*, *PLIN1*, *PNPLA3*, *SDR16C5*, *TMEM135*) and PPAR signaling pathway (*ADIPOQ*, *AQP7*, *aP2*, *PLIN1*, *PPARγ*) enriched in up regulation gene set (Fig. S1f; Table S5). We also observed the *ADIPOQ* gene displaying significantly differential RNA expression (*P* = 7.65E−14) among High and Low. Accumulating evidence indicated that mRNA m^6^A modification could mediate transcription regulation [12, 48]. Thus, to investigate whether m^6^A contributes to translation regulation in *longissimus* muscle, we overlapped the genes significantly difference both in the level of m^6^A modification and RNA expression between High and Low. Finally, we found 70 target co-differential genes (Fig. 3d, e), including *ADIPOQ* and *SFRP1*, which were related to several pathways such as PPAR signaling pathway (*ADIPOQ*, *AQP7*, *aP2*) (Table S4). *SFRP1* gene has been reported that inhibits the Wnt/β-catenin signaling pathway, regulating the adipogenesis both in human and murine [49]. *ADIPOQ* gene is expressed specifically in adipose tissue [50], which exhibited higher expression in porcine fat tissues including subcutaneous fat and intramuscular fat than LDM in the same population [23]. To illustrate the mechanism of m^6^A modification on regulating the adipogenesis, we then chosen the hub gene *ADIPOQ* with remarkably methylated and RNA expression co-differential for further investigation (Fig. 3f, g).

### *ADIPOQ* promotes adipogenesis of preadipocytes in vitro

**Fig. 4:**
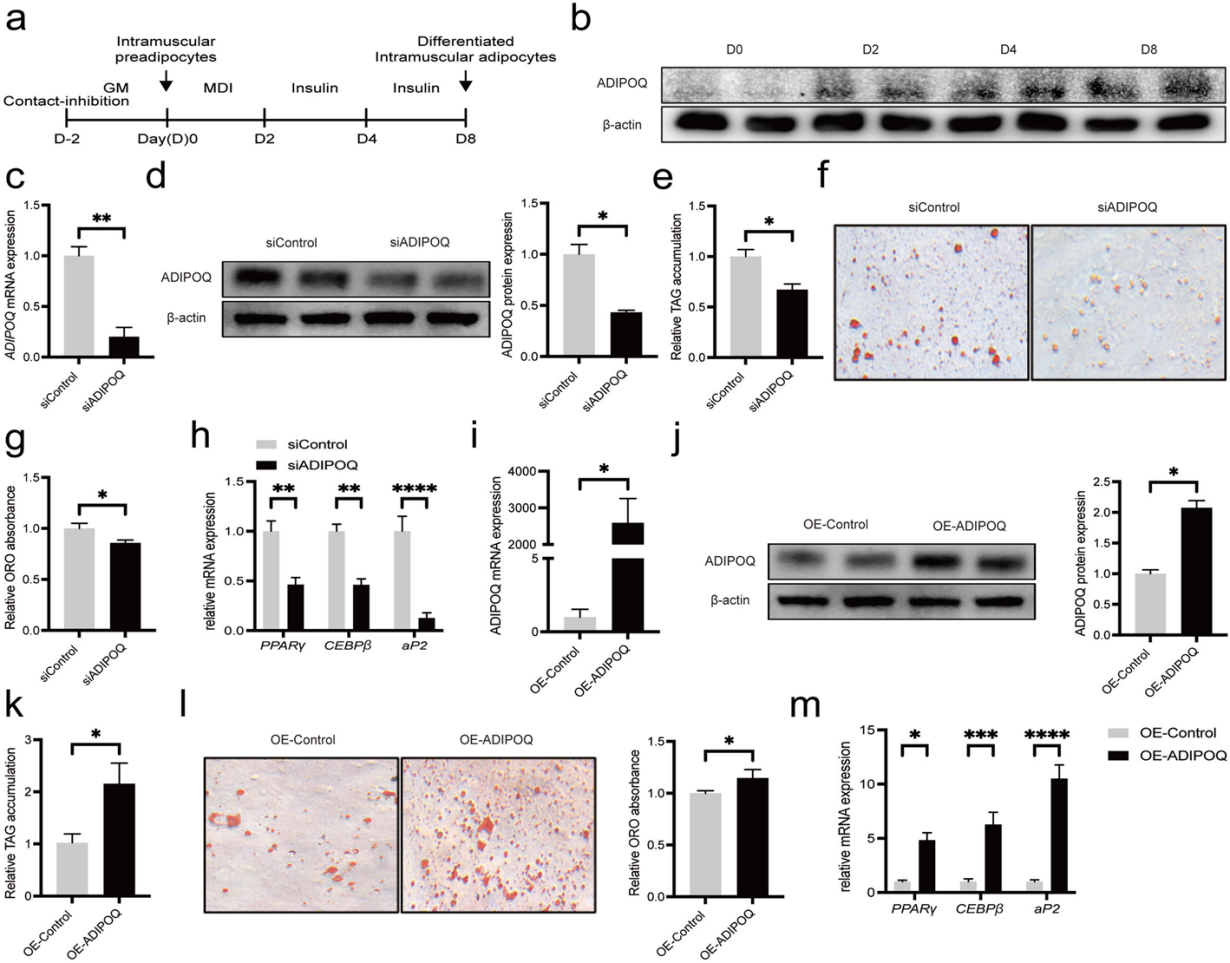
*ADIPOQ* promote adipogenesis of preadipocytes in vitro. **a** Workflow of porcine intramuscular adipocytes inducing in vitro. **b** Protein levels of ADIPOQ in intramuscular preadipocytes at 0, 2, 4 and 8 d during adipogenesis. **c** The mRNA levels of *ADIPOQ* (48 h) after siRNA transfection of porcine intramuscular preadipocytes, *n* = 3. **d** The protein expression levels of ADIPOQ (48 h) after siRNA transfection of porcine intramuscular preadipocytes. **e–g** TAG content and Oil Red O staining of *siADIPOQ* at 8 d after adipogenic induction, *n* = 3. **h** RT-qPCR of *PPARγ*, *CEBPβ* and *aP2* of *siADIPOQ* at 8 d after adipogenic induction, *n* = 3. **i** The mRNA expression levels of *ADIPOQ* after overexpression *ADIPOQ* (48 h), *n* = 3. **j** The protein expression levels of ADIPOQ after overexpression ADIPOQ (48 h). **k**,** l** TAG content and Oil Red O staining of ADIPOQ-overexpression at 8 d after adipogenic induction, *n* = 3. **m** RT-qPCR of *PPARγ*, *CEBPβ* and *aP2* of ADIPOQ overexpression at 8 d after adipogenic induction, *n* = 3. ^*^P < 0.05, ^**^P < 0.01, ^***^P < 0.001, ^****^P < 0.0001

To re-validate whether *ADIPOQ* gene regulates adipogenesis, intramuscular preadipocytes were isolated for adipogenic differentiation by the standard IBMX, dexamethasone, and insulin (MDI) cocktail (Fig. 4a). The lipid accumulation and mRNA expression levels of adipogenic genes (*PPARγ*, *CEBPβ* and *aP2*) were significantly increased after MDI induction (Fig. S2a, b). Simultaneously, the expression of ADIPOQ mRNA and protein were significantly increased from d 0 to 8 (Fig. S2c; Fig. 4b).

Previous research had indicated that interference with *ADIPOQ* gene expression could inhibit the differentiation of porcine preadipocytes [42]. Thus, we established siRNA and overexpression plasmid to address the function of *ADIPOQ* in the process of adipogenic differentiation in our work. Not surprisingly, mRNA expression and protein level of *ADIPOQ* were significantly inhibited after siRNA interference at d 8 (Fig. 4c, d). Meanwhile, lipid accumulation of siADIPOQ was remarkably decreased according to triacylglycerol (TAG), Oil Red O staining and adipogenic genes (*PPARγ*, *CEBPβ* and *aP2*) mRNA expression (Fig. 4e–h). Next, we observed the overexpression of *ADIPOQ* in porcine intramuscular preadipocytes cell could significantly increase the levels of its mRNA expression and protein (Fig. 4i, j), promoting lipid accumulation (Fig. 4k–m). On the basis of above results, we concluded that *ADIPOQ* was expressed at the later stage of induction and promoted porcine intramuscular preadipocyte differentiation and lipid accumulation.

### mRNA m^6^A modification can promote *ADIPOQ* expression

**Fig. 5:**
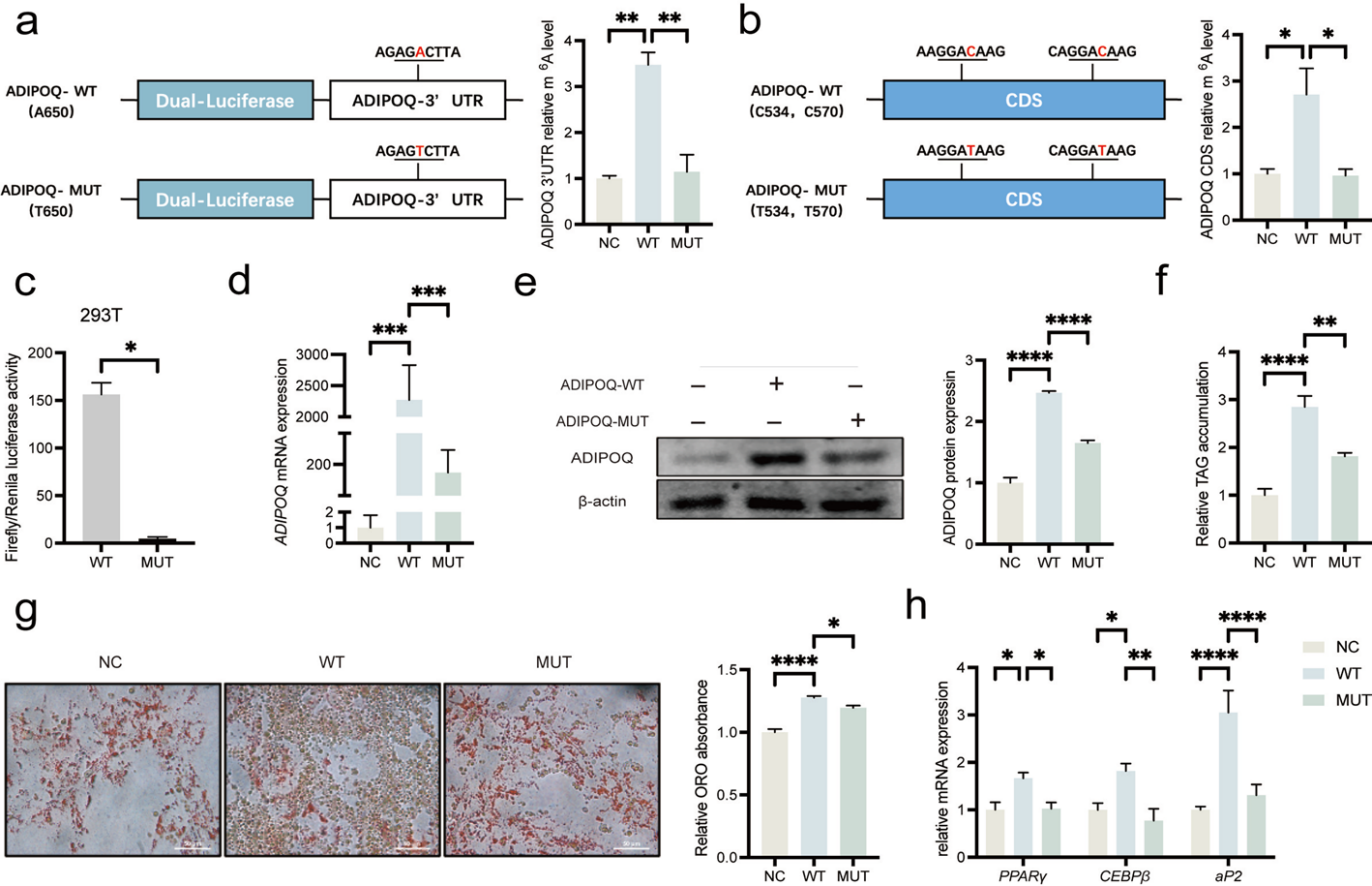
*ADIPOQ* promotes adipogenesis of preadipocytes in a m^6^A-dependent manner. **a** m^6^A-IP-qPCR analysis of ADIPOQ-3’UTR WT or MUT (A to T mutation) in 293 T cells, *n* = 3. **b** m^6^A-IP-qPCR analysis ADIPOQ-CDS WT or MUT (C to T mutation) in porcine intramuscular preadipocytes, *n* = 3. **c** Relative luciferase activity of WT or MUT of ADIPOQ-3’UTR in 293 T cells, *n* = 3. **d** The mRNA expression levels of *ADIPOQ* of porcine intramuscular preadipocytes with NC, WT or MUT of ADIPOQ-CDS, *n* = 3. **e** The protein expression levels of ADIPOQ of porcine intramuscular preadipocytes with NC, WT or MUT of ADIPOQ-CDS. **f** and** g** TAG content and Oil Red O staining of porcine intramuscular preadipocytes with NC, WT or MUT of ADIPOQ-CDS at 8 d after adipogenic induction, *n* = 3. **h** RT-qPCR of *PPARγ*, *CEBPβ* and *aP2* of porcine intramuscular preadipocytes with NC, WT or MUT of ADIPOQ-CDS at 8 d after adipogenic induction, *n* = 3. ^*^P < 0.05, ^**^P < 0.01, ^***^P < 0.001, ^****^P < 0.0001

Although we acquired that *ADIPOQ* could promote the lipid accumulation in intramuscular preadipocytes, the role of m^6^A modification in *ADIPOQ* remain unclear [42, 47]. To further explore the function of mRNA m^6^A modification on *ADIPOQ* expression, we firstly scanned the transcript to uncover the m^6^A sites of *ADIPOQ* gene based on the RRACH conversed feature. Three potential m^6^A sites including one in 3’UTR (AGACT, chr13:124,645,333–124,645,337) and two in CDS (GGACA, chr13:124,644,484–124,644,488; GGACA chr13:124,644,520–124,644,524*)* were found in the longest *ADIPOQ* transcript ENSSSCT00000047495 (Fig. 3f). To explore the role of m^6^A modification in 3’UTR and CDS of *ADIPOQ*, we constructed the dual-luciferase reporter plasmid and adenovirus vector with mutation in 3’UTR (ADIPOQ-3’UTR-MUT) and CDS (ADIPOQ-CDS-MUT), respectively (Fig. 5a, b; Table S6). Analysis of m^6^A-IP-qPCR found that m^6^A methylation levels of ADIPOQ-CDS-WT and ADIPOQ-3’UTR-WT were higher than ADIPOQ-CDS-MUT and ADIPOQ-3’UTR-MUT, respectively (Fig. 5a). Luciferase assays results indicated that mutation of *ADIPOQ* 3’UTR significantly decreased the luciferase activity in 293 T cells (Fig. 5c). Consistently, the mRNA expression and protein level of ADIPOQ in ADIPOQ-CDS-WT IMF cells were also higher than ADIPOQ-CDS-MUT (Fig. 5d, e). We also found ADIPOQ-CDS-MUT decreases lipid accumulation (Fig. 5f, g) and adipocyte differentiation-related gene expression including *PPARγ*, *CEBPβ* and *aP2*, relative to ADIPOQ-CDS-WT (Fig. 5h). Taken together, we concluded that the m^6^A modification of *ADIPOQ* in 3’UTR and CDS could both promote its expression.

### YTHDF1 mediates the regulation of *ADIPOQ* in an m^6^A-dependent manner

**Fig. 6:**
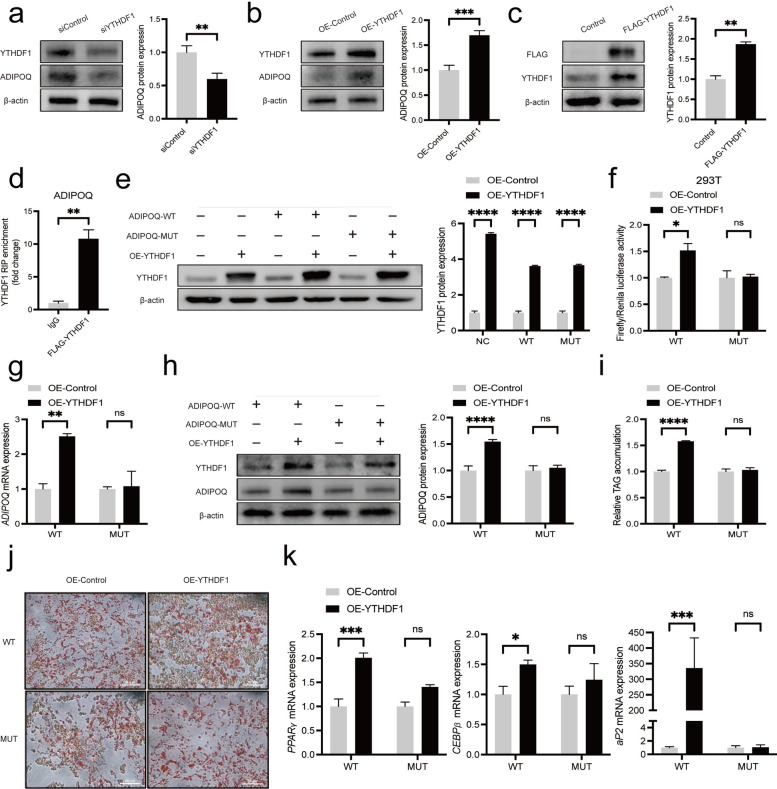
YTHDF1 regulate the translation of *ADIPOQ* in IMF cells. **a** The protein levels of ADIPOQ in porcine intramuscular preadipocytes transfected with siControl or siYTHDF1 (48 h). **b** The protein levels of ADIPOQ after overexpression YTHDF1 (48 h). **c** The protein levels of YTHDF1 of porcine intramuscular preadipocytes transfected with control or YTHDF1-FLAG plasmid (48 h). **d** RIP analysis of the interaction of *ADIPOQ* with FLAG in porcine intramuscular preadipocytes transfected with YTHDF1-FLAG plasmid. Enrichment of *ADIPOQ* with FLAG was measured by qPCR and normalized to input. **e** The protein levels of YTHDF1 of 293 T cells transfected with WT or MUT of ADIPOQ-3’UTR or YTHDF1 overexpression plasmid (48 h). **f** Relative luciferase activity of WT or MUT of ADIPOQ-3’UTR or YTHDF1 overexpression in 293 T cells, *n* = 3. **g** The mRNA expression levels of *ADIPOQ* of porcine intramuscular preadipocytes with WT or MUT of ADIPOQ-CDS or YTHDF1-overexpression plasmid (48 h), *n* = 3. **h** The protein expression levels of *ADIPOQ* of porcine intramuscular preadipocytes with WT or MUT of ADIPOQ-CDS or YTHDF1-overexpression plasmid (48 h). **i** and** j** TAG content and Oil Red O staining of porcine intramuscular preadipocytes with WT or MUT of ADIPOQ-CDS or YTHDF1 overexpression plasmid at 8 d after adipogenic induction, *n* = 3. **k** RT-qPCR of *PPARγ*, *CEBPβ* and *aP2* of porcine intramuscular preadipocytes with WT or MUT of ADIPOQ-CDS or YTHDF1 overexpression plasmid at 8 d after adipogenic induction, *n* = 3. ^*^P < 0.05, ^**^P < 0.01, ^***^P < 0.001, ^****^P < 0.0001

We then explored the mechanism about how m^6^A modification regulated *ADIPOQ* expression. YTHDF1 was reported to promote translation of m^6^A methylated transcripts [51]. Regarding the m^6^A sites in 3’UTR or CDS could promote the translation of *ADIPOQ*, we assumed that *ADIPOQ* is the target of YTHDF1. Thus, we performed YTHDF1 knockdown and overexpressing experiments to identify whether it could regulate *ADIPOQ* expression. Not surprisingly, YTHDF1 knockdown decreased ADIPOQ protein expression (Fig. 6a), while YTHDF1 overexpression increased ADIPOQ protein expression (Fig. 6b). RIP-qPCR assay revealed that *ADIPOQ* interacted with YTHDF1-FLAG, which confirmed that *ADIPOQ* is the target of YTHDF1 (Fig. 6c, d). To further explore whether YTHDF1 targets and recognizes the *ADIPOQ* mRNA m^6^A modification site, we transferred YTHDF1 overexpression plasmid into ADIPOQ-3’UTR-WT (or MUT) and ADIPOQ-CDS-WT (or MUT) cells, respectively. Overexpression of YTHDF1 increased luciferase activity and ADIPOQ protein level in ADIPOQ-3’UTR-WT 293 T cells, but no change in ADIPOQ-3’UTR-MUT (Fig. 6e, f) cells. Similarly, overexpressing YTHDF1 increased the mRNA and protein expression of ADIPOQ in ADIPOQ-CDS-WT IMF cells but no change in ADIPOQ-CDS-MUT cells (Fig. 6g, h). Moreover, we also observed overexpressing YTHDF1 increases lipid accumulation (Fig. 6i, j) and adipocyte differentiation-related gene expression including *PPARγ*, *CEBPβ* and *aP2* (Fig. 6k) in ADIPOQ-CDS-WT but not in ADIPOQ-CDS-MUT. Collectively, these results together suggest YTHDF1 promotes the translation of hub gene *ADIPOQ* by recognizing m^6^A sites in both 3’UTR and CDS.

## Discussion

In this work, we performed the m^6^A-seq of LDM from a unique heterogenous swine population to investigate the underlying mechanism of mRNA m^6^A modification regulating IMF deposition. We revealed that hub gene *ADIPOQ* could promote its mRNA translation in an m^6^A-YTHDF1-dependent manner, providing novel evidence of m^6^A methylation regulating adipogenesis.

Fat deposition is highly relevant to human health [52, 53], uncovering the mechanism porcine intramuscular adipogenesis is better for understanding gene regulation underlying the fat deposition of corresponding tissues in humans. Accumulating evidences demonstrate that m^6^A modification is involving in adipogensis pathway [17–19]. Although previous finding has been revealed that m^6^A modification of *MTCH2* promotes adipogenesis in LDM when comparing obese Asian domesticated Jinhua pig and lean Western commercial pig, these results was still limited because of the selected validation gene merely obtain from top 10 methylation in Jinhua breed [15]. In this study, we possessed different hallmark from previous studies in that a unique swine population was used [15, 54], and found that some individuals exhibits a large variation of IMF content. More importantly, IMF content was negatively related to the mRNA m^6^A level across the High and Low group (*P* < 0.01), indicating a potential role of m^6^A in intramuscular fat deposition, which was consistent to previous study.

To explore the underlying role and mechanism of m^6^A in porcine intramuscular fat, we performed a large sample size of MeRIP data (*n* = 10 per group), which allowed us to explore more significant differential m^6^A modification sites. Tao et al. uncovered 5,872 and 2,826 m^6^A peaks respectively, in the porcine muscle and adipose tissue transcriptomes [54]. Here, we identified a total of 23,250 m^6^A peaks in this population, to our knowledge, it is largest m^6^A data set in procine intramuscular fat. Besides, the consensus motif sequence RRACH in our study was consistent with previous work. mRNA m^6^A sites were enriched around stop codons, sharing a smiliar distribution to those of human, mice and plants [55–57]. Taken together, using larger sample size and stringent m^6^A calling parameters, our results allow a reliable picture of the mRNA m^6^A epi-transcriptome in porcine skeletal muscles.

To uncover which key genes regulate adipogenesis in m^6^A-dependent manner, we performed gene co-expression network using WGCNA to explore the biologically relevant associations between phenotype and module [35]. Finally, we uncovering 70 modules among 19 high expression RNA input data, including 12 hub genes, were significantly corelated with IMF content and m^6^A modification level. Emerging evidences have indicated that WGCNA could reveal potential candidate gene in affecting the IMF content of Duroc [58] and Italian Large White pigs [59]. Thus, we overlapped RNA expression differential and m^6^A methylated differential genes, discovering 70 co-differential genes. We further found 2 hub gene *ADIPOQ* and *SFRP1* including in co-differential gene set. These results largely advanced our knowledge towards co-expression networks in IMF deposition.

In this work, we found *ADIPOQ* gene displayed remarkably differential both in RNA expression (*P* = 7.65E−14) and m^6^A methylation (*P* = 5.09E−05). *ADIPOQ* has been identified as candidate gene for the metabolic syndrome and T2DM by genome wide associated study [60, 61]. Previous work also indicated *ADIPOQ* exhibited higher expression in both intramuscular fat and subcutaneous fat than LDM in the same swine population [23]. Consistently, previous study provided supportive evidence for silencing of *ADIPOQ* efficiently suppresses preadipocyte differentiation in porcine [42]. By establishing the lipogenesis model in vitro*,* we revalidated the *ADIPOQ* gene could promote the adipogenesis of porcine preadipocyte. We further found mRNA m^6^A modification could promote the expression of *ADIPOQ* and lipid accumulation by constructing the dual-luciferase reporter plasmid and adenovirus vector in 3’UTR and CDS, respectively.

Various m^6^A binding proteins, especially YTHDF family, have been proved their functions in different aspects, such as RNA translation, splicing, export or degradation [62, 63]. YTHDF1 selectively recognizes m^6^A in cytosolic mRNAs, recruiting initiation factor eIF3 to facilitate mRNA translation [51]. YTHDF2 brings m^6^A-modified translatable mRNAs to mRNA decay sites (e.g., P-bodies), and recruiting CC chemokine receptor 4-negative regulator of transcription complex to trigger mRNA deadenylation [9, 64]. YTHDF3 promotes mRNA translation in synergy with YTHDF1 and accelerated decay of m^6^A-containing mRNAs through interaction with YTHDF2. Accumulating evidences suggest YTHDF1 promote RNA expression via recognizing mRNA m^6^A site [65, 66]. YTHDF1 interacting with *MTCH2* mRNA to enhance translation of its protein in porcine intramuscular preadipocytes [15, 67]. Thus, we here have conducted interference and overexpression YTHDF1 to confirm its function. Not surprising, we observed YTHDF1 promoting the translation of hub gene *ADIPOQ*, confirming that *ADIPOQ* was a target of YTHDF1 through m^6^A-IP and RIP experiments.

## Conclusions

In conclusion, our study characterized the m^6^A modification genes which were potentially involved in regulating IMF deposition. Furthermore, we presented a novel regulatory mechanism of IMF deposition via the m^6^A-YTHDF1-ADIPOQ axis, highlighting the critical role of mRNA m^6^A modification of the hub gene in IMF adipogenesis.

## Supplementary Information

**Additional file 1: Fig. S1.** RNA expression analysis of MeRIP-seq input data. **a** and **b** PCA and heatmap of highly expression genes among 20 and (**c** and **d**) 19 samples (excluded L96), respectively. **e** Volcano plot of RNA differential expression gene (*P*-adjust < 0.05 and fold change > 1.5), *DSPP* gene in the dotted box for extremely outlier of the figure (log_2_foldchange = − 25.3; *P*-adjust = 1.36E−17)

**Additional file 2: Fig. S2.** Establishment of lipogenesis model in vitro. **a** Oil Red O staining of porcine intramuscular preadipocytes at 0, 2, 4 and 8 d after adipogenic induction, *n* = 3. **b** RT-qPCR of *ADIPOQ* of porcine intramuscular preadipocytes at 0, 2, 4 and 8 d after adipogenic induction, *n* = 3. **c** RT-qPCR of *PPARγ*, *CEBPβ* and *aP2* of porcine intramuscular preadipocytes at 0, 2, 4 and 8 d after adipogenic induction, *n* = 3. ^*^P < 0.05, ^**^P < 0.01, ^***^P < 0.001, ^****^P < 0.0001

**Additional file 3:** **Table S1.** 70 genes in MEdarkturquoise module

**Additional file 4:** **Table S2.** m^6^A differential modified genes

**Additional file 5:** **Table S3.** RNA expression differential genes

**Additional file 6:** **Table S4.** Methylated and RNA expression co-differential genes

**Additional file 7:** **Table S5.** KEGG and GO analysis of gene set from Table S1-S4

**Additional file 8: Table S6.** m^6^A modification mutation site of *ADIPOQ* cDNA in CDS and 3′ UTR

## Abbreviations

ACE2: Angiotensin converting enzyme 2
ADIPOQ: Adiponectin
aP2: Fatty acid binding protein 4
AQP7: Aquaporin 7
BAM: Binary Alignment/Map format
BMP2: Bone morphogenetic protein type 2
CDS: Coding sequence
CEBPα: CCAAT enhancer binding protein alpha
CEBPβ: CCAAT enhancer binding protein beta
CIDEC: Cell death inducing DFFA like effector c
DMEM: Dulbecco’s modified eagle medium
FFAR4: Free fatty acid receptor 4
GO: Gene ontology
HACD2: 3-hydroxyacyl-CoA dehydratase 2
IBMX: 3-Isobutyl-1-methylxanthine
IgG: Immunoglobulin G
IMF: Intramuscular fat
IP: Immunoprecipitation
ITIH3: Inter-alpha-trypsin inhibitor heavy chain 3
KEGG: Kyoto Encyclopedia of Genes and Genomes
LDM: Longissimus dorsi muscle
m^6^A: N6-methyladenosine
MDI: Dexamethasone, and insulin
MTCH2: Mitochondrial carrier homolog 2
MUT: Mutation
OD: Optical density
PLIN1: Perilipin 1
PMSF: Phenylmethanesulfonyl fluoride
PNPLA3: Patatin like phospholipase domain containing 3
PPAR: Peroxisome proliferator activated receptor
PPARγ: Peroxisome proliferator activated receptor gamma
Qpcr: Real-time quantitative PCR
RIP: RNA immunoprecipitation
SDR16C5: Short chain dehydrogenase/reductase family 16C member 5
SFRP: Secreted frizzled-related protein 1
SH3PXD2B: SH3 and PX domain-containing protein 2B
SNCG: Synuclein gamma
SORL1: Sortilin related receptor 1
TAG: Triacylglycerol
T2DM: Type 2 diabetes mellitus
TMEM135: Transmembrane protein 135
UTR: Untranslated region
UNC93A: Unc-93 homolog A
WGCNA: Weighted correlation network analysis
WT: Wide type
YTHDF1–3: YT521‑B homology domain family proteins 1–3
