# Supplementary figures and images for: Profiling of N6-methyladenosine methylation in porcine longissimus dorsi muscle and unravelling the hub gene ADIPOQ promotes adipogenesis in an m6A-YTHDF1–dependent manner
Source: J Anim Sci Biotechnol. 2023 Apr 6;14:50. doi: 10.1186/s40104-023-00833-4 (PMC10077699; doi:10.1186/s40104-023-00833-4)

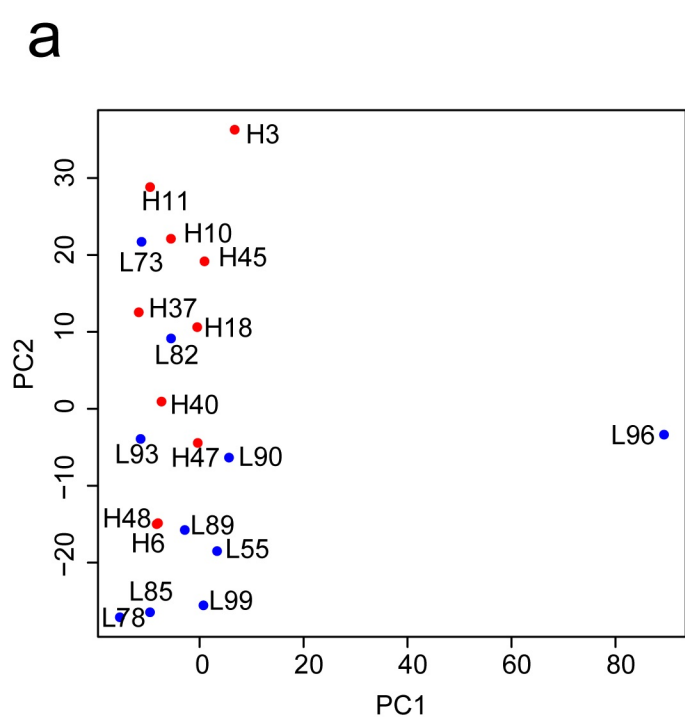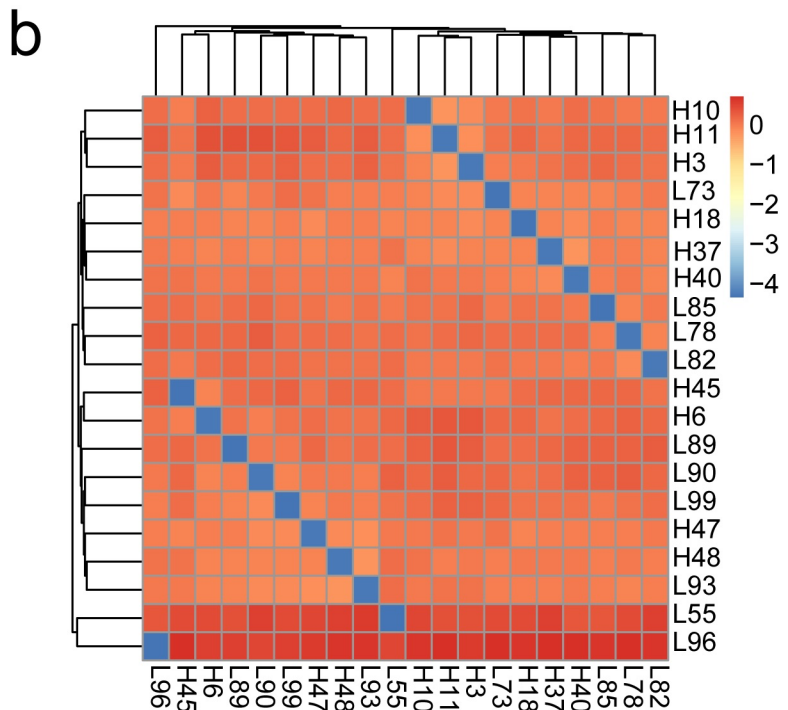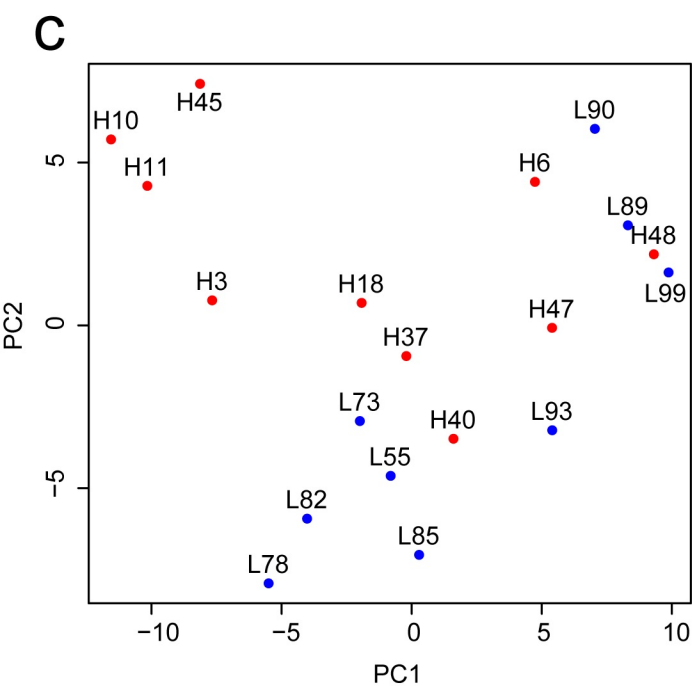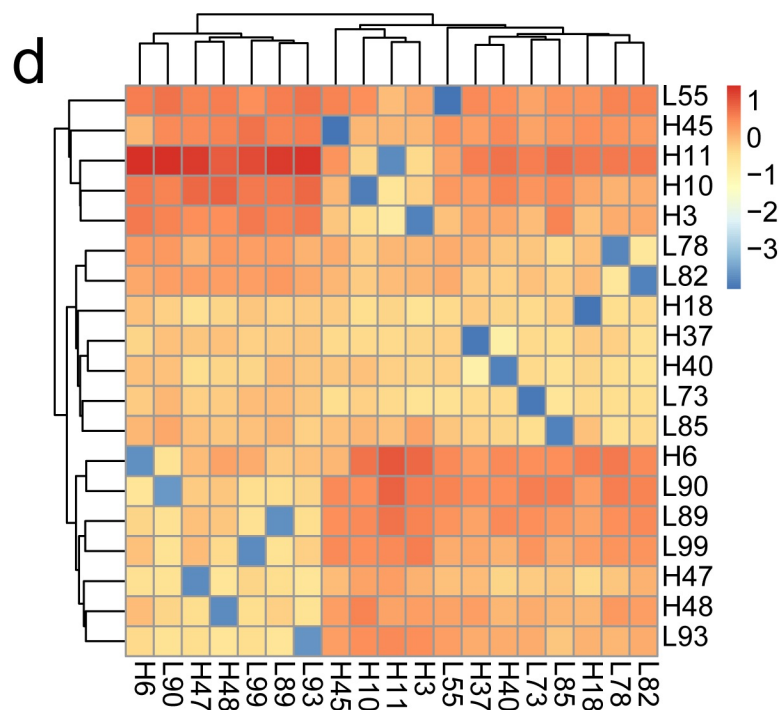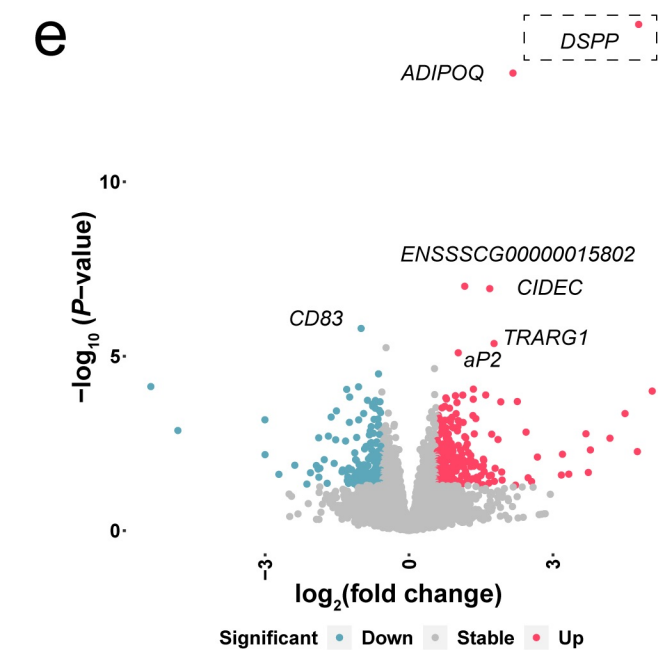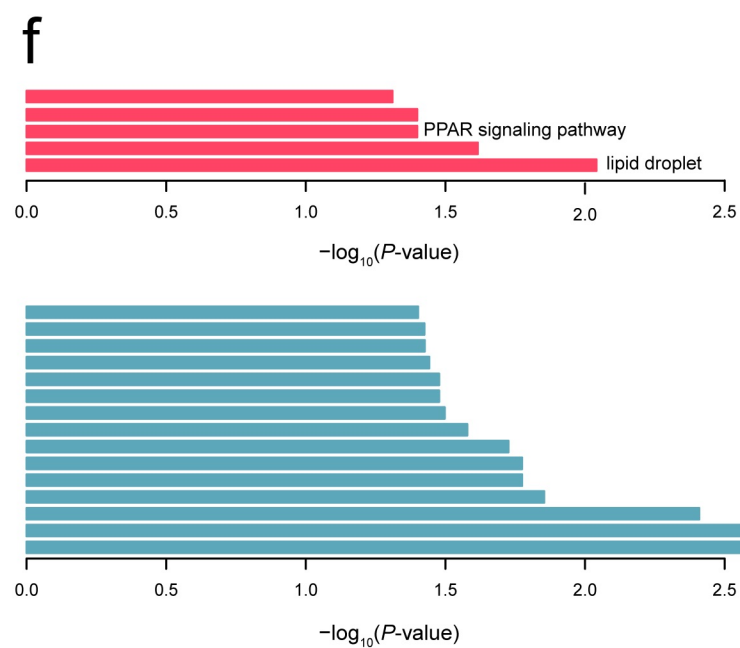

Supplement: Supplementary file 1 — Additional file 1: Fig. S1. RNA expression analysis of MeRIP-seq input data. a and b PCA and heatmap of highly expression genes among 20 and (c and d) 19 samples (excluded L96), respectively. e Volcano plot of RNA differential expression gene (P-adjust < 0.05 and fold change > 1.5), DSPP gene in the dotted box for extremely outlier of the figure (log2foldchange = − 25.3; P-adjust = 1.36E−17) [file 40104_2023_833_MOESM1_ESM.pdf]

a

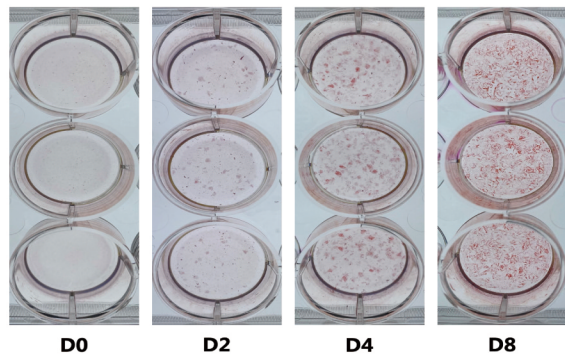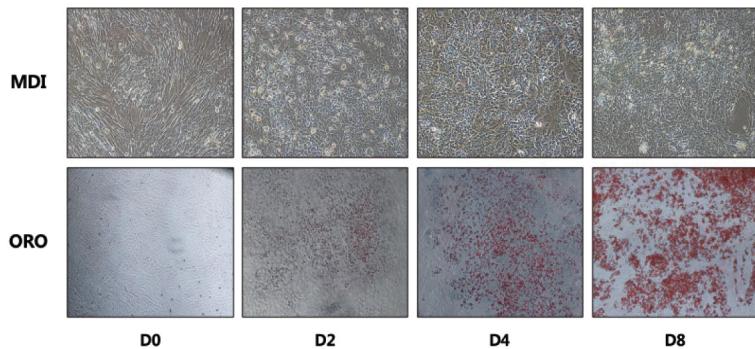

b

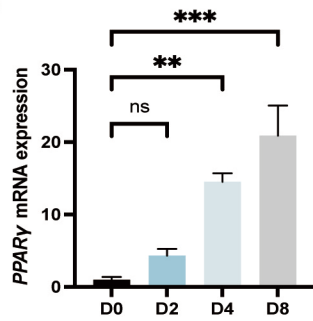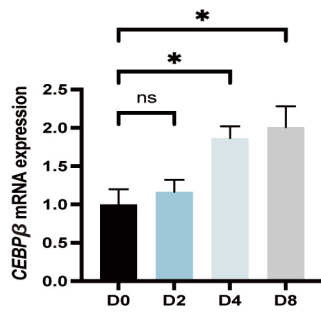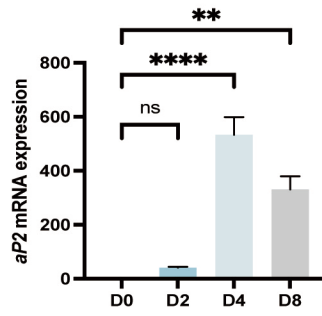

c

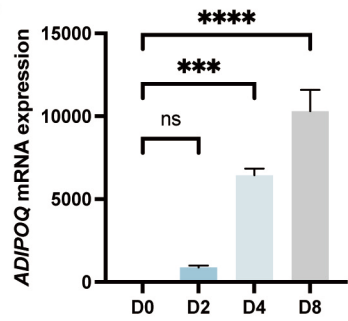

Supplement: Supplementary file 2 — Additional file 2: Fig. S2. Establishment of lipogenesis model in vitro. a Oil Red O staining of porcine intramuscular preadipocytes at 0, 2, 4 and 8 d after adipogenic induction, n = 3. b RT-qPCR of ADIPOQ of porcine intramuscular preadipocytes at 0, 2, 4 and 8 d after adipogenic induction, n = 3. c RT-qPCR of PPARγ, CEBPβ and aP2 of porcine intramuscular preadipocytes at 0, 2, 4 and 8 d after adipogenic induction, n = 3. *P < 0.05, **P < 0.01, ***P < 0.001, ****P < 0.0001 [file 40104_2023_833_MOESM2_ESM.pdf]
