# Supplementary material for: Profiling of N6-methyladenosine methylation in porcine longissimus dorsi muscle and unravelling the hub gene ADIPOQ promotes adipogenesis in an m6A-YTHDF1–dependent manner
Source: J Anim Sci Biotechnol. 2023 Apr 6;14:50. doi: 10.1186/s40104-023-00833-4 (PMC10077699; doi:10.1186/s40104-023-00833-4)
Supplement: Supplementary file 8 — Additional file 8: Table S6. m6A modification mutation site of ADIPOQ cDNA in CDS and 3′ UTR [file 40104_2023_833_MOESM8_ESM.docx]

**Supplementary Table 6. m^6^A modification mutation site of ADIPOQ cDNA in CDS and 3’ UTR**

| **m^6^A site** | **Sequence** |
| --- | --- |
| ADV4-ADIPOQ-CDS-WT m^6^A sites | TGGCAAATTCCACTGCAACATTCCTGGGCTGTACTACTTCTCCTTCCACATCACGGTCTACTTGAAGGATGTGAAGGTCAGCCTCTACAAGAA**GGACA**AGGCTGTACTCTTCACCTACGACCAGTACCA**GGACA**AGAATGTGGACCAGGCCTCTGGCTCTGTGCTCCTCTATCTGGAGAAGGGGGACCAAGTCTGGCTCC |
| ADV4-ADIPOQ-CDS-MUT m^6^A sites | TGGCAAATTCCACTGCAACATTCCTGGGCTGTACTACTTCTCCTTCCACATCACGGTCTACTTGAAGGATGTGAAGGTCAGCCTCTACAAGAA**GGATA**AGGCTGTACTCTTCACCTACGACCAGTACCA**GGATA**AGAATGTGGACCAGGCCTCTGGCTCTGTGCTCCTCTATCTGGAGAAGGGGGACCAAGTCTGGCTCC |
| ADIPOQ-3’UTR with wild-type m^6^A site | TGAATTTCTGGGCCCACTGTGTTTCCTCAGGTTCATACCAATTCTATGAAGTACATAGGAAAGATCTTCTCTTGGGGATGAG**AGACT**TAAGTGAACGCCCAAGGTTACACAGACACTCAATGGCAGAGTTAGAAGTCAAACACAGGGCTGTGGACTTTTTTCAGCATACTACGCC |
| ADIPOQ-3’UTR with mutant m^6^A site | TGAATTTCTGGGCCCACTGTGTTTCCTCAGGTTCATACCAATTCTATGAAGTACATAGGAAAGATCTTCTCTTGGGGATGAG**AGTCT**TAAGTGAACGCCCAAGGTTACACAGACACTCAATGGCAGAGTTAGAAGTCAAACACAGGGCTGTGGACTTTTTTCAGCATACTACGCC |

ADV4, adenovirus vector type 4.
